# Radial Structure Scaffolds Convolution Patterns of Developing Cerebral Cortex

**DOI:** 10.3389/fncom.2017.00076

**Published:** 2017-08-15

**Authors:** Mir Jalil Razavi, Tuo Zhang, Hanbo Chen, Yujie Li, Simon Platt, Yu Zhao, Lei Guo, Xiaoping Hu, Xianqiao Wang, Tianming Liu

**Affiliations:** ^1^School of Environmental, Civil, Agricultural and Mechanical Engineering, College of Engineering, University of Georgia Athens, GA, United States; ^2^Cortical Architecture Imaging and Discovery Lab, Department of Computer Science and Bioimaging Research Center, University of Georgia Athens, GA, United States; ^3^School of Automation, Northwestern Polytechnic University Xi'an, China; ^4^Department of Small Animal Medicine & Surgery, College of Veterinary Medicine, University of Georgia Athens, GA, United States; ^5^Biomedical Imaging Technology Center, Emory University Atlanta, GA, United States

**Keywords:** neuroimaging, radial structure, radial convolution pattern, computational modeling

## Abstract

Commonly-preserved radial convolution is a prominent characteristic of the mammalian cerebral cortex. Endeavors from multiple disciplines have been devoted for decades to explore the causes for this enigmatic structure. However, the underlying mechanisms that lead to consistent cortical convolution patterns still remain poorly understood. In this work, inspired by prior studies, we propose and evaluate a plausible theory that radial convolution during the early development of the brain is sculptured by radial structures consisting of radial glial cells (RGCs) and maturing axons. Specifically, the regionally heterogeneous development and distribution of RGCs controlled by Trnp1 regulate the convex and concave convolution patterns (gyri and sulci) in the radial direction, while the interplay of RGCs' effects on convolution and axons regulates the convex (gyral) convolution patterns. This theory is assessed by observations and measurements in literature from multiple disciplines such as neurobiology, genetics, biomechanics, etc., at multiple scales to date. Particularly, this theory is further validated by multimodal imaging data analysis and computational simulations in this study. We offer a versatile and descriptive study model that can provide reasonable explanations of observations, experiments, and simulations of the characteristic mammalian cortical folding.

## Introduction

**Figure 1:**
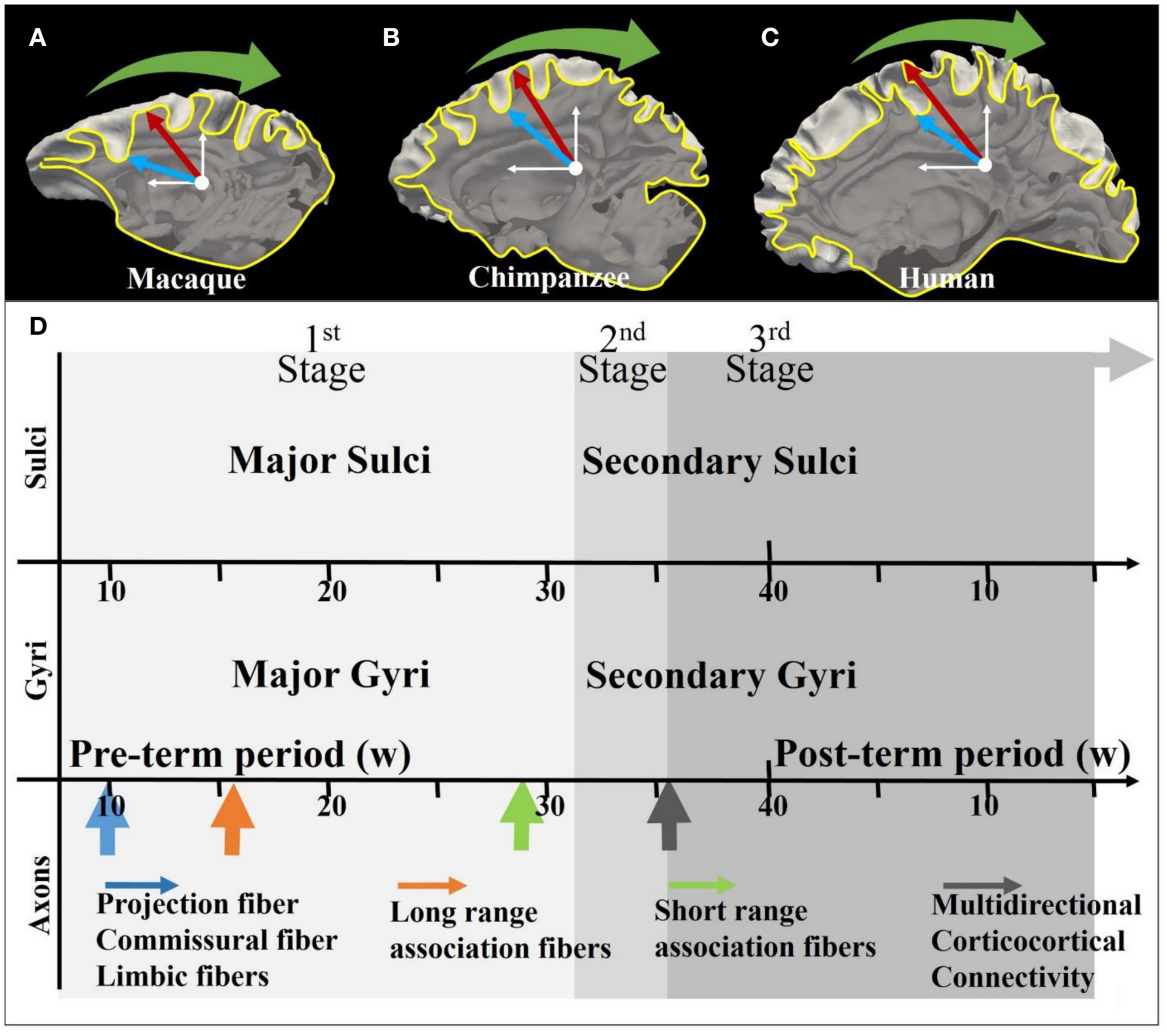
**(A–C)** Illustration of the coordinate system on mature macaque, chimpanzee, and human cortical surface. The white axes indicate the stereoscopic coordinate system on sagittal planes. The yellow curves are the intersection lines of the cortical surface and the sagittal planes. The red arrows and blue arrows, respectively indicating gyri and sulci, point along radial directions on the sagittal plane. The green arrows suggest the circumferential directions; **(D)** Joint time course of gyrogenesis and axonogenesis of the human brain. The x-axis represents the timeline (see White et al., 2010 for explanations of the time segments). The arrows in the axon panel indicate the boosting time point of the specific type of axons. A detailed version of the time course chart can be found in Supplemental Figure 1.

Elaborate convolution of the cerebral cortex is one of the most unique and prominent characteristics of the mammalian brain, and is to a certain extent maintained across species. Generally, correspondence between major gyri and sulci (radial direction convolution pattern, see Figure 1) is well-preserved across primate species (Li et al., 2016) while the level of shape complexity and curvature (tangential direction convolution pattern, see Figure 1) is known to vary (Toro and Burnod, 2005; Zhang et al., 2017). For instance, even though the cortical convolution complexity increases from the macaque brain, to the chimpanzee brain and the human brain (Figures 1A–C), convolution patterns in the radial direction, such as in the central gyri and sulci, can still be consistently identified (Chen et al., 2013). The formation of such convolution patterns could be induced by multiple brain development processes, such as neurogenesis and axonogenesis, and the coupling of gyrogenesis and these processes have been reported in many works (Chi et al., 1977; Huang et al., 2009; White et al., 2010; Takahashi et al., 2012; Dubois et al., 2013). Numerous promising hypotheses in line with these observations have been put forward to discover the underlying mechanisms of cortical convolution.

For example, cranial constraints were suggested to be an external factor that causes inward convolution (Le Gros Clark, 1945; Chen et al., 2010), such that extending cortices could be held in a limited space. In the differential growth hypothesis, the driving mechanism for instability and convolution is differential growth on cellular bases (Caviness, 1975; Brown et al., 2002; Cartwright, 2002). Recent experimental and computational findings suggested that growth rate of the outer layers is higher compared with the inner layers of the brain (Bayly et al., 2013; Ronan et al., 2013; Razavi et al., 2015b; Tallinen et al., 2016).

Another school of hypotheses suggested that axon maturation also play role on the gyrification process (Van Essen, 1997; Sur and Rubenstein, 2005; Nie et al., 2012; Holland et al., 2015; Zhang et al., 2016, 2017). Figure 1D illustrates the coupling of gyrogenesis and axonogenesis across the time course, as was suggested to be divided into three stages (White et al. 2010). The first stage features the emergence of relatively stable primary fissures (Toro and Burnod, 2005) and projection fibers and long-range association fibers together with dominating radial glial cell fibers (Huang et al., 2009; Takahashi et al., 2012). In the secondary and tertiary stages, numerous subtle convolutions are gradually appended to the framework of the first-stage convolutions, introducing multi-directional (e.g., the tangential direction) variations (Chi et al., 1977; Toro and Burnod, 2005), which are accompanied by the emergence of U shape fibers (the green arrow in Figure 1) and multi-directional corticocortical connections (black arrow in Figure 1D) (see detailed explanations in Supplemental Materials).

However, a comprehensive understanding of how these processes cooperatively interact in order to give rise to experimentally-observed brain development still remains to be elucidated (Van Essen, 1997; Monuki and Walsh, 2001; Grove and Fukuchi-Shimogori, 2003; Sur and Rubenstein, 2005; Rakic, 2006). For example, it still remains elusive as to why the primary cortical convolution pattern across subjects within each species is highly correlated and consistent rather than random, and what factors account for this consistency as regulators. Recently, a growing number of genetic studies have reported the discovery of potential fundamental molecular regulators for cortical morphology (Beck et al., 1995; Rakic, 1995; Leighton et al., 2001; Gaudillière et al., 2004; Konishi et al., 2004; Stahl et al., 2013; Sun and Hevner, 2014). However, more studies are required to explore exactly how those genetic fundamental factors take effects to play a part in phenotypic characterization.

Inspired by prior theories (Rakic, 1995; Nie et al., 2012; Borrell and Götz, 2014), we propose that radial glial cells (RGCs) and neuronal axons (defined here as radial structures) are the determining regulators of radial convolution patterns in the early stage of gyrogenesis. To this end, we incorporate recent multidisciplinary discoveries and observations, i.e., genotype-based experiments and phenotype-based measurements, as well as computational simulations, into a single theoretical framework. In this paper, we define the radial direction as the direction pointing from the core of the brain to the cortex (Figures 1A–C), and the convolution pattern in this direction as gyral (convex) or sulcal (concave). Based on these definitions, our theory puts forward the following two hypotheses: (i) RGCs regulate radial convolution patterns of the cerebral cortex (Rakic, 1995; Borrell and Götz, 2014) and (ii) the interplay between maturing axons and RGCs regulates convex folding patterns.

The rest of the paper is organized as follows. In the Materials and Methods Section, we introduce multi-modal data analysis techniques and our computational modeling analysis. In Results Section, the proposed theory is validated by this joint analysis of multi-modal imaging data and simulation experiments, in which we test a variety of possible configurations via computation by tuning experimentally validated parameters. Computational results are quantitatively compared with those from literature reports as well as observations and measurements on our MRI and histology data.

## Materials and methods

### Brain imaging and preprocessing

#### Diffusion-weighted MRI and T1-weighted MRI

**Figure 2:**
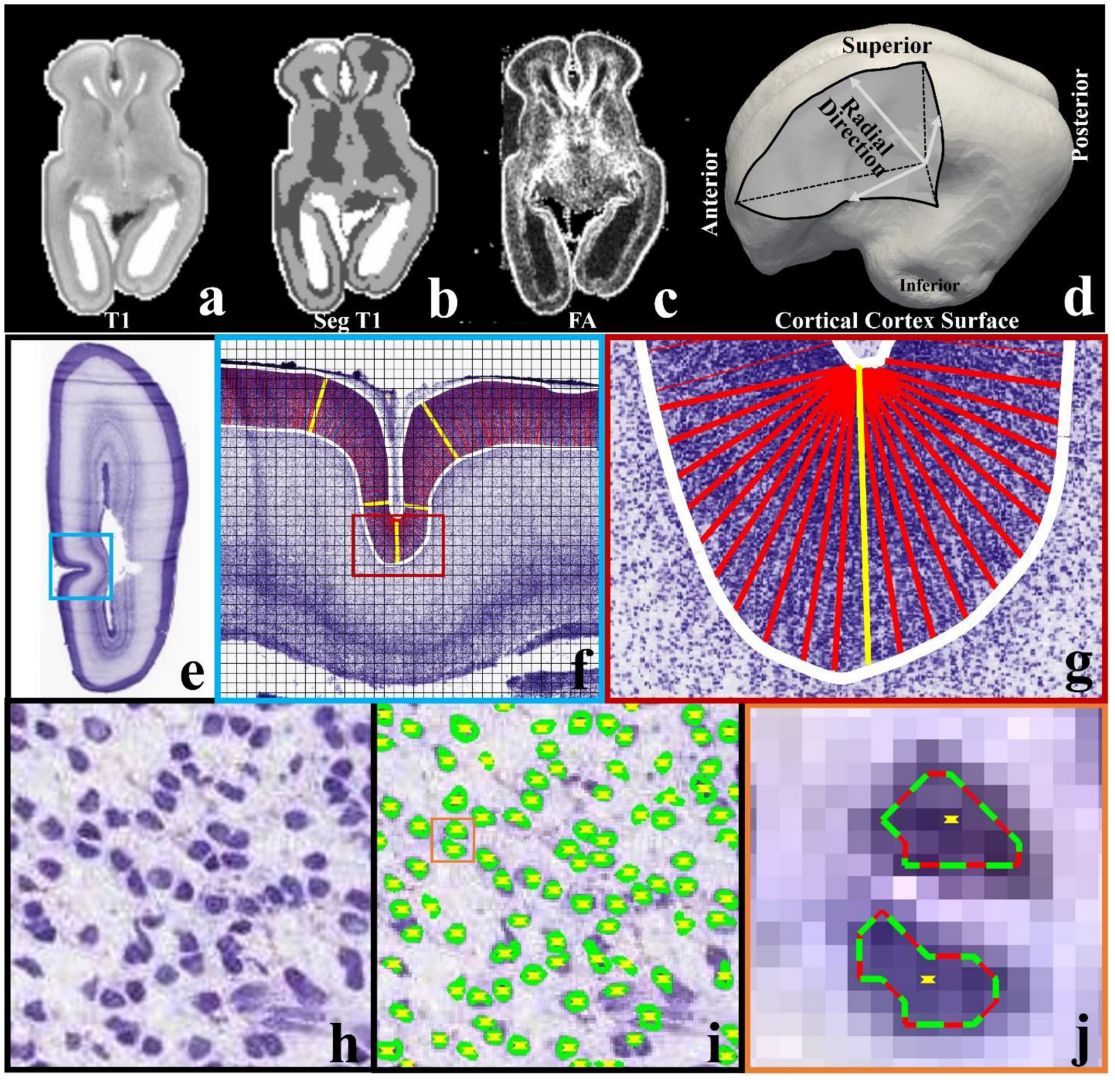
**(a)** A slice of T1-weighted MRI data; **(b)** the slice in **(a)** has been segmented into three types of tissues, i.e., white matter (dark), gray matter (gray), and non-brain fluid (white); **(c)** FA map of the same slice; **(d)** cortical triangular mesh surface reconstructed on the boundary between brain tissue (white matter and gray matter) and non-brain fluid in **(b)**. Arrows indicate the radial directions; **(e)** a slice from a 21 pcw fetal brain sectioned in the coronal plane and stained for Nissl; **(f)** An enlarged view of the regions highlighted by the blue frame in **(e)**. The slice is cut into 128-by-128 pixel image blocks (black chessboard), on which methods in Arteta et al. (2012) are adopted to identify neurons. White curves are manually depicted cortical plate boundaries. Columns within the cortical plate and perpendicular to the boundaries are illustrated by dense red bars. The yellow bars illustrate some example columns; **(g)** an enlarged illustration of columns in the region highlighted by the red frame in **(f)**; **(h)** one image block in **(f)**. Purple blobs are neuronal cells; **(i)** Neuron identification results of the image block in **(h)**. Green/red curves indicate extremal neuron boundaries. Yellow dots indicate neuron centers; **(j)** an enlarged view of neuron identification results in the orange color frame in **(i)**.

A 21pcw *ex vivo* prenatal brain from the Allen Brain Institute (http://www.brain-map.org/) is used. Detailed dataset description can be found in the BrainSpan Developing Human Brain Imaging whitepaper (http://www.brainspan.org/docs.html). For convenience, important imaging configuration and parameters are listed here. Imaging is done on a 3T MRI scanner (Siemens Medical Solutions, Erlangen, Germany) using a customized solenoid coil. Diffusion tensor imaging scans are performed using a 3D diffusion-weighted steady state free precession (DW-SSFP) sequence. Imaging parameters are listed as follows: *TR* = 24.5 ms, TE = 18.76 ms, isotropic spatial resolution 0.4 mm, bandwidth 150 Hz/px, 8 non-diffusion-weighted volumes and 44 diffusion-weighted volumes. For the T1-weighted MRI data, optimal imaging parameters for gray/white contrast-to-noise ratios (CNR) per-unit-time is obtained based on data within the range of likely optimal parameters and using Bloch equation-based parameter-estimation^1^. T1-weighted MRI data provided has been skull-stripped (Figure 2a). FSL's FAST is used to segment white matter, gray matter and non-brain fluid (gray, dark, and white regions in Figure 2b). We use the white matters and the gray matters as the brain mask. The cortical surface (Figure 2c) is reconstructed on the brain mask using in-house methods based on the marching cube algorithm (Liu et al., 2008).

Streamline tractography fibers are reconstructed via Trackvis (http://www.trackvis.org/) in the individual space. The angular threshold is set to be 40° as suggested in Takahashi et al. (2012). Instead of using a fractional anisotropy (FA) threshold as the stopping criteria of tractography, the T1-weighted brain mask linearly registered (FSL's FLIRT) to the FA map is applied to terminate tract tracking (Takahashi et al., 2010, 2012; Vishwas et al., 2010).

#### Histology

Nissl-stained brain sections are obtained from a 21pcw fetus (http://www.brainspan.org). The right hemisphere is sectioned into 20 μm thick sections in the coronal plane. A total of 81 sections are chosen for annotation in the reference atlas. Figure 2e shows one section for example. Details can be found in documents for BrainSpan^2^.

Neuron detection on Nissl-stained sections is implemented *via* the automated method listed in Arteta et al. (2012). Generally, the method works on 128-by-128 pixel Nissl-stained image blocks (see Figure 2f) within a machine learning scheme. It requires only a couple of image blocks with simple dot annotation for training. A dot is placed inside each cell. In the training stage, models are learned within a structured SVM framework. The method uses a maximally stable extremal region detector (MSER) (Matas et al., 2004) to find a number of candidate regions, the non-overlapping subset with high similarity to the annotated regions can then be selected. The learned model is applied to testing image blocks and returns the predicted neuronal cell centers and extremal boundaries (see Figures 2h–j).

Neuron number counting is performed based on the neuronal cell identification results. In-plane columns (width: 40 μm; length: the cortical plate depth. See Figures 2f,g) are used as counting boxes (similar to the methods in Hilgetag and Barbas, 2005). To produce the columns, we manually extract the cortical plate boundaries (white curves in Figure 2f). The columns are rooted on the superior boundary with equal spacing (around 120 μm) between any two adjacent columns. In flat regions, the columns are set to be perpendicular to the boundaries. In U-turn regions, such as the sulcal fundus in Figure 2g, the perpendicular constraint is relaxed. If a center of a neuron is within a distance *r* of the column, it will be considered as being covered by the column. In this paper *r* is set to be 3 pixels (around 3 μm) such that even the smallest neurons are counted.

### Computational model

**Figure 3:**
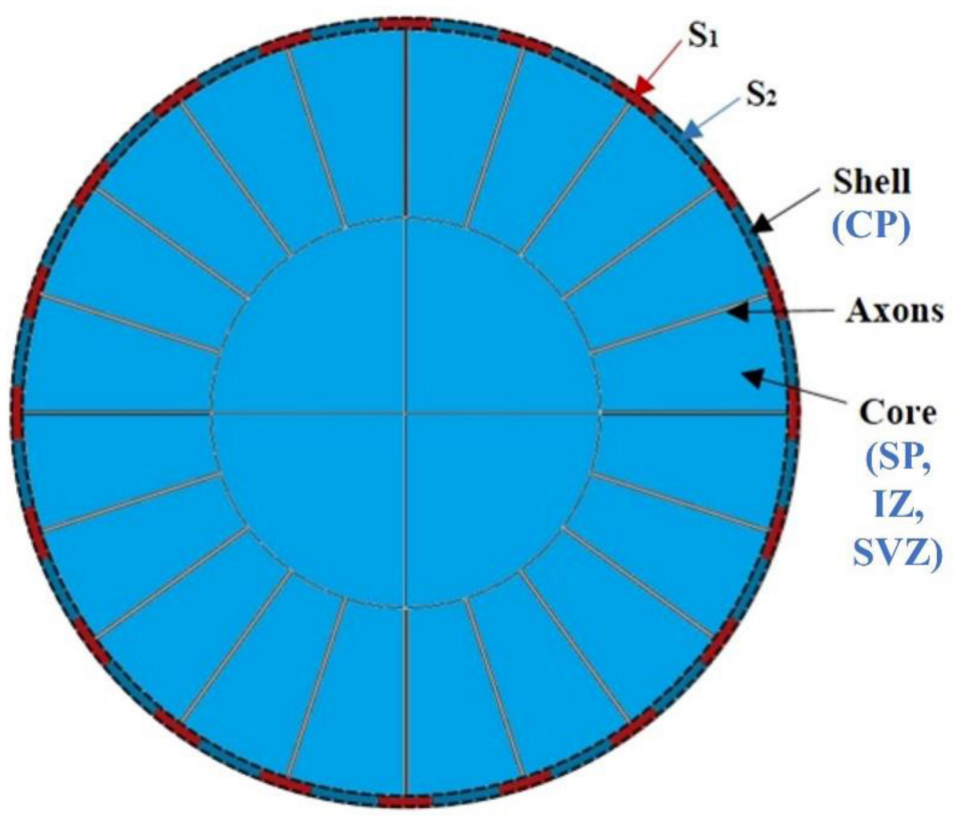
An idealized 2D computational model of the brain. CP, cortical plate; SP, subplate, intermediate zone; SVZ, sub-ventricular zone.

Recently, computational modeling is used widely to unravel mechanism of the brain convolution from mechanical view (Bayly et al., 2013; Budday et al., 2014; Tallinen et al., 2014, 2016; Razavi et al., 2015a). The prominent advantage of computational modeling is in its strong ability to fine-tune the configuration of models such that we can effectively screen through a variety of scenarios to validate or repudiate various theoretical hypotheses. Therefore, a computational modeling approach bridges the gap between “dynamic” theoretical hypotheses and “static” observations and analyses, which is another major interest of this study. To do so, a two-dimensional (2D) circular model consisting of a bilayer soft tissue (Figure 3) is constructed in this study in order to investigate the radial structure effects and potential mechanisms of cortical convolution in the first stage of brain development. The shell of the model represents the developing cortical plate and the core is a simple organization of the subplate, intermediate zone, and sub-ventricular zone. The developing radial structure (RGCs and axons) are modeled by tubes radially distributed and connecting the core and shell (Bayly et al., 2013; Tallinen et al., 2014).

In our model, the variable parameters of interest are the growth speed of the shell (g_s_), core (g_c_), and radial tubes (g_f_) as well as their stiffness (μ_s_, μ_c_, and μ_f_). In some cases, different growth speeds are assigned to different shell regions s_1_ and s_2_ (the variation of parameters and reasons are discussed in the Results Section). In order to focus on in-plane brain bifurcation and eliminate longitudinal effects, we assume that the deformation field after growth is only a function of the radius in this circular bilayer model. Given the structural symmetry of the model, only the first quadrant is shown in later figures. In general, the elastic deformation of a living soft tissue yields little volume change; therefore, a nonlinear response of the material can be described by an incompressible hyperelastic material (Jin et al., 2011; Li et al., 2011; Cao et al., 2012; Razavi and Wang, 2015; Razavi et al., 2016). Here a simple and common model based on an isotropic nonlinear neo-Hookean material is implemented (Tallinen et al., 2014; Razavi et al., 2015a,b).

(1) $$\begin{array}{r} \text{W}=\frac{\mu }{2}\left({\lambda_{\text{r}}}^{2}+{\lambda_{\theta }}^{2}+{\lambda_{\text{z}}}^{2}-3\right) \end{array}$$

where *W* is the energy density, μ is the shear modulus and λ_*r*_, λ_*O*_, and λ_*z*_ are the radial, circumferential and axial principal stretches, respectively. All simulations are carried out in the commercial finite element software ABAQUS. The outer shell of the brain model is allowed to self-contact. More information about the model can be found in the Supplemental Materials.

## Results and discussions

Based on the experimental observations and also validation by the computational simulations, we present our findings in three parts. In Section Regional Growth Heterogeneity on Cortex Drives Convex and Concave Patterns Convolution, it is showed that regional growth heterogeneity on cortex drives convex and concave convolution patterns. In Section Axons Serve as Regulators of Convex Radial Convolution, we show that axons serve as regulators of convex radial convolution. In section Interplay of Neurogenesis and Axonogenesis Acts as a Convex (Gyral) Convolution Pattern Regulator, we show that interplay of the former two factors acts as a convex convolution pattern regulator.

### Regional growth heterogeneity on cortex drives convex and concave convolution patterns

In this section, we study how neurogenesis takes effects on gyrogenesis from imaging and histology data and computational modeling results. Generally, RGCs with lower levels of Trnp1 could generate basal progenitors (BPs), also known as intermediate progenitor cells (IPCs), and basal radial glial cells (bRGCs). BPs produce neurons while bRGCs provide additional guiding structures to induce faster neuron migration; this finally results in considerable radial and lateral cortical expansion, i.e., the convex folding pattern suggested in Götz and Huttner (2005) and Stahl et al. (2013). Therefore, at the cellular level, a distribution difference of RGCs regulates the radial expansion of the cortical plate by controlling the amount of migrating neurons. Our multi-modal data analysis and computational modeling in this section are conducted based on this model.

We firstly use imaging and histology data to quantify the relationship between neuron numbers and radial cortical folding patterns, and the relationship between features of short radial structures (or RGCs, a combination of aRGCs and bRGCs) such as their orientations and radial cortical folding patterns. Notably, when reporting our results, we use the term radial structures (sometimes we use “all” radial structures) to represent the mixture of RGCs and maturing axons in the radial direction, and short radial structures to represent RGCs.

#### Imaging data evidence

##### Short radial structures vs. radial convolution patterns

**Figure 4:**
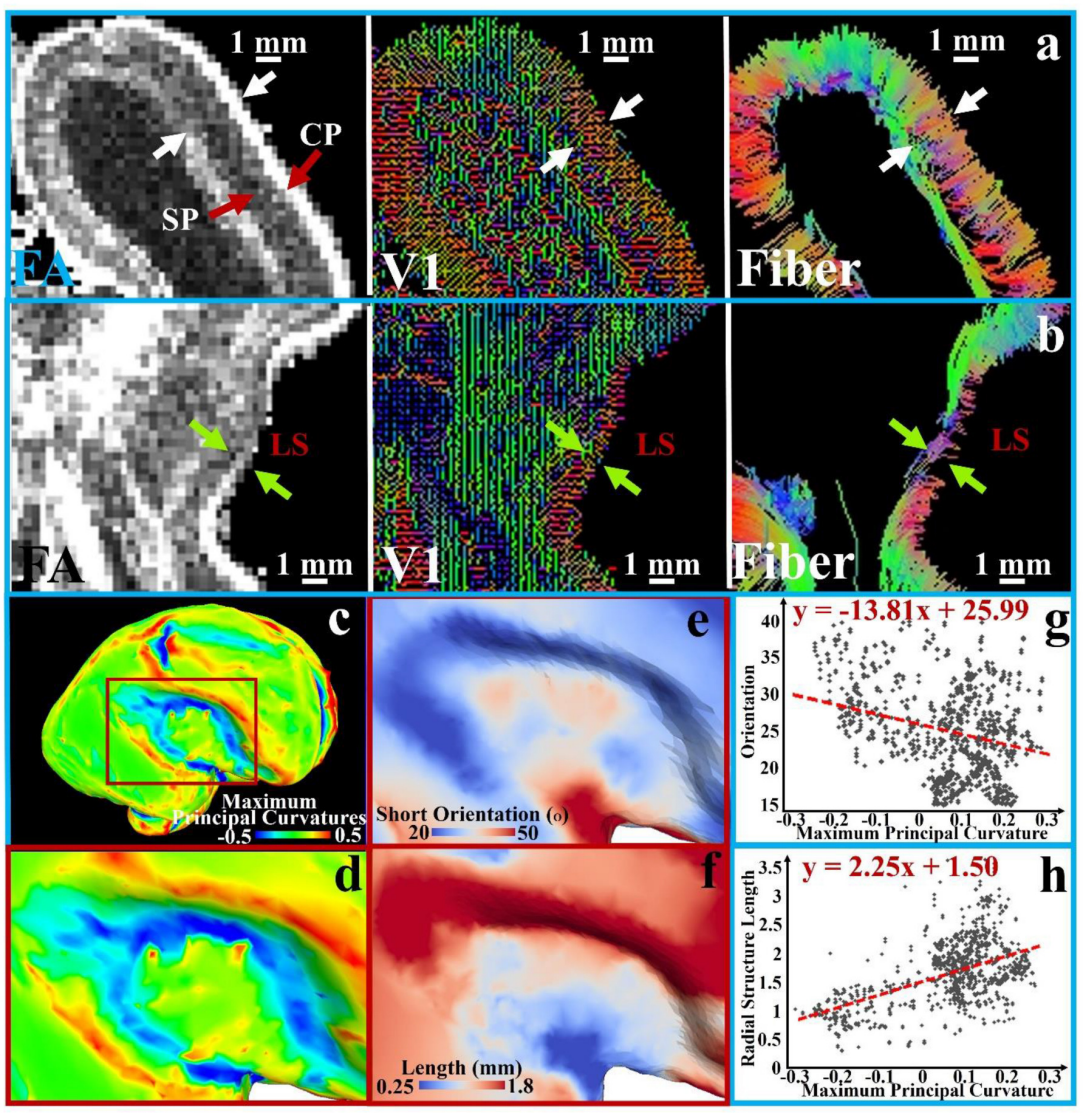
A transverse section is extracted from FA, V1, and tractography fiber maps of the 21 pcw fetus diffusion tensor imaging (DTI) data. An enlarged view of the occipital part of the brain is shown in **(a)** and LS part is in **(b)**; white and green arrows in pairs indicate the thickness of subplate (SP) and cortical plate (CP); **(c)** The brain surface with maximum principal curvatures is reconstructed; **(d)** An enlarged view of the LS region with surface maximum principal curvatures; **(e)** The angles between average orientation of the short radial structures and the normal directions of the local surface regions on the LS regions; **(f)** The lengths of short radial structures are mapped to the LS regions. Short subcortical structures are defined as the streamline fibers shorter than 5 mm; **(g–h)** The measurements [angles in **(e)** and length in **(f)**] of sampled vertices in the LS regions are shown against the maximum principal curvatures in the scatter plots. A 1st order linear trend is estimated and illustrated by red dashed lines.

The aforementioned assumption is illustrated in Figure 4 based on analysis and observation of DTI data of *ex vivo* 21 pcw fetal brains from the BrainSpan: Atlas of the Developing Human Brain (http://developinghumanbrain.org). By applying a threshold to the tractography streamline fibers tracts (where the length is less than 5 mm), we observe in the tractography fiber maps from Figures 4a,b and Supplemental Figure 2 that the short radial structures between the subplate (SP) and the cortical plate (CP) are distributed across the entire cortical regions. Short radial structures have been suggested to be a mixture of RGC bundles (Takahashi et al., 2012). In V1 maps (Figures 4a,b and Supplemental Figure 2), a similar observation is reproduced based on the primary tensor eigenvector direction.

The difference between the lateral sulcus (LS) and other convex regions can be noticed in those DTI based measurements. In Figure 4a, clear boundaries of the SP and CP are identified in the FA map and V1 maps while they are ambiguous in the LS region from the same section as shown in Figure 4b. We quantitatively map the length of the short radial structures to the cortical plate surface (Figure 4f shows an enlarged view of LS region), and also measure and map the angle between the average normal direction from the surface patch and the average short radial structure orientations from the patch (the LS region in Figure 4e). An angle of zero degrees indicates that the short radial structure radially contacts the surface while an angle of 90° indicates that the structure is parallel to the surface. The maximum principal curvatures of the surface in Figures 4c,d are used as a reference, in which convex regions have positive values while concave ones have negative values. The relationships between maximum principal curvatures and the angel/length measured based on a couple of sample vertices in this region are shown in Figures 4b,g. Short radial structures in convex regions perpendicularly connect the cortical surface while those beneath the concave regions are more likely to be parallel to the surface. The length of the short radial structures of a surface is directly proportional to the convexity of that surface, while an inversely proportional relationship is found for their orientations. In summary, the concave LS region has fewer radially oriented fibers and the length of those radial structures are shorter, suggesting that the cortical plate in concave regions is not only thinner, but also that fewer RGCs are generated in this region. These observations are consistent with what had been reported in literature (Stahl et al., 2013).

##### Neuron number vs. radial convolution patterns.

**Figure 5:**
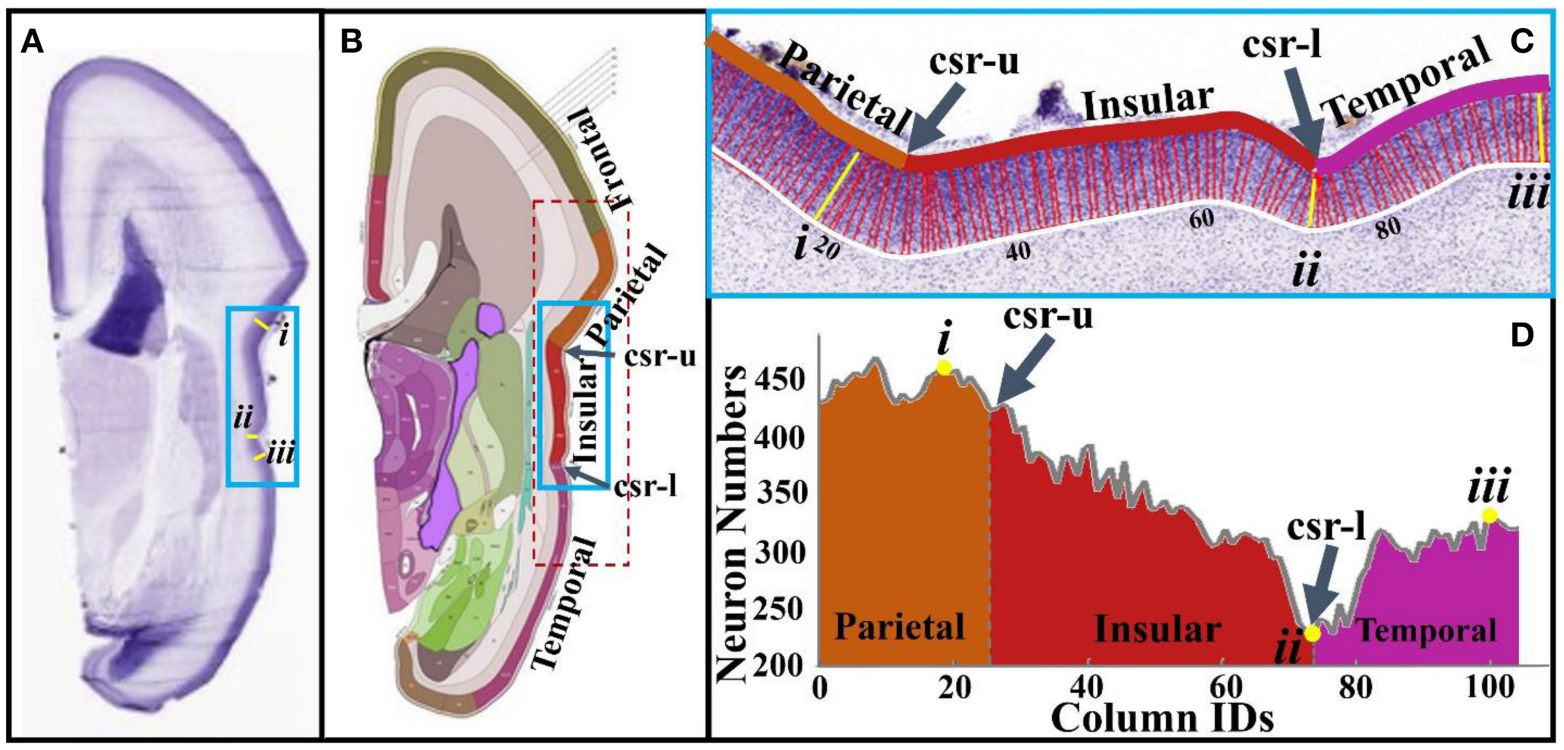
**(A)** A slice from a 21 pcw fetal brain sectioned in the coronal plane and stained for Nissl; **(B)** The plane atlas of the 21 pcw fetal cerebrum; the blue frame highlights insular regions; **(C)** An enlarged view of insular regions highlighted by the blue frame in **(A,B)**. The boundaries of the cortical plane (color and white curves) are manually depicted. Columns, i.e., the red and yellow colored bars between boundaries of the cortical plate, are used as neuron number counting boxes. These columns are perpendicular to the white boundary. The width of each column is 40 μm. Arabic numberals indicate the column IDs; **(D)** Neuron numbers of the columns in **(C)**. Lower-case Roman numberals indicate local maxima (i, iii) and minima (ii). csr, circular sulcus of Reil; csr-u, upper limiting sulcus; csr-l, lower limiting sulcus.

To conduct neuron number counting, columns are produced in the cortical plate according to the methods in Section Brain Imaging and Preprocessing (see Figure 5C). The accuracy of neuron detection on this dataset is evaluated in Supplemental Materials. The reference atlas (Figure 5B) from http://atlas.brain-map.org/ is used to segment the cortical plate into different anatomical regions. Using this basis, the cortical plate columns inside the red dashed frame in Figure 5B are separated into the concave cortex (insular) group and convex cortex (non-insular) group. Statistically, a right tail *t*-test is used to test the alternative hypothesis that the neuron number in convex columns on average is greater than that of the one in concave columns, with a significance level (α) being set to 0.05. The *P*-value of the test is 5.05 × 10^−6^, suggesting that there truly are more neurons in convex regions than in concave regions. Figures 5C,D show neuron numbers in insular regions (blue frames in Figures 5A,B). It can be observed that, moving from the parietal to the insular lobe, neuron numbers decrease until reaching a minimum value (#ii column) at the lower limiting sulcus (csr-l). Then, the number of neurons rapidly increases quickly moving to the columns of the temporal lobe. Another example using the calcarine fissure can be found in the Supplemental Figure 5. These results are in line with those of Hilgetag and Barbas (2005), that gyral regions have a significantly larger number of neurons and thicker laminar than non-gyral regions.

In summary, these observations and measurements on imaging and histology data provide direct support for the model that the distribution of RGCs in the human fetal brain is regionally heterogeneous. Those regions with fewer RGCs (short radial structures in DTI data) have fewer neurons migrating to the cortical plate, which may produce a thin cortical plate and concave convolution patterns in the radial direction. These observation and measurements are thus extremely useful clues for evaluating the following computational models.

#### Computational models

In accordance with the observations from brain imaging and histology data, we conduct computational experiments using the morphological model introduced in Section Computational Model. Here we set the growth speed of the shell (g_s_), identified by the red mesh in Figure 3, to be faster than that of the core (g_c_), the gray colored regions beneath the shell. Heterogeneous growth speeds amongst laminae had been reported to be a possible critical factor in generating convolution (Caviness, 1975; Brown et al., 2002; Cartwright, 2002). From an energetic viewpoint, the increase of the growth ratio creates a rise in the residual stress up to the critical value in the circumferential direction of the model, thereby triggering instability and creasing in the model in order to release the energy. Residual stress has been observed in growing soft biological tissues and is believed to play a crucial role in morphogenesis and regulation of the material properties of biological systems (Ben Amar and Goriely, 2005; Li et al., 2012; Bayly et al., 2014).

**Figure 6:**
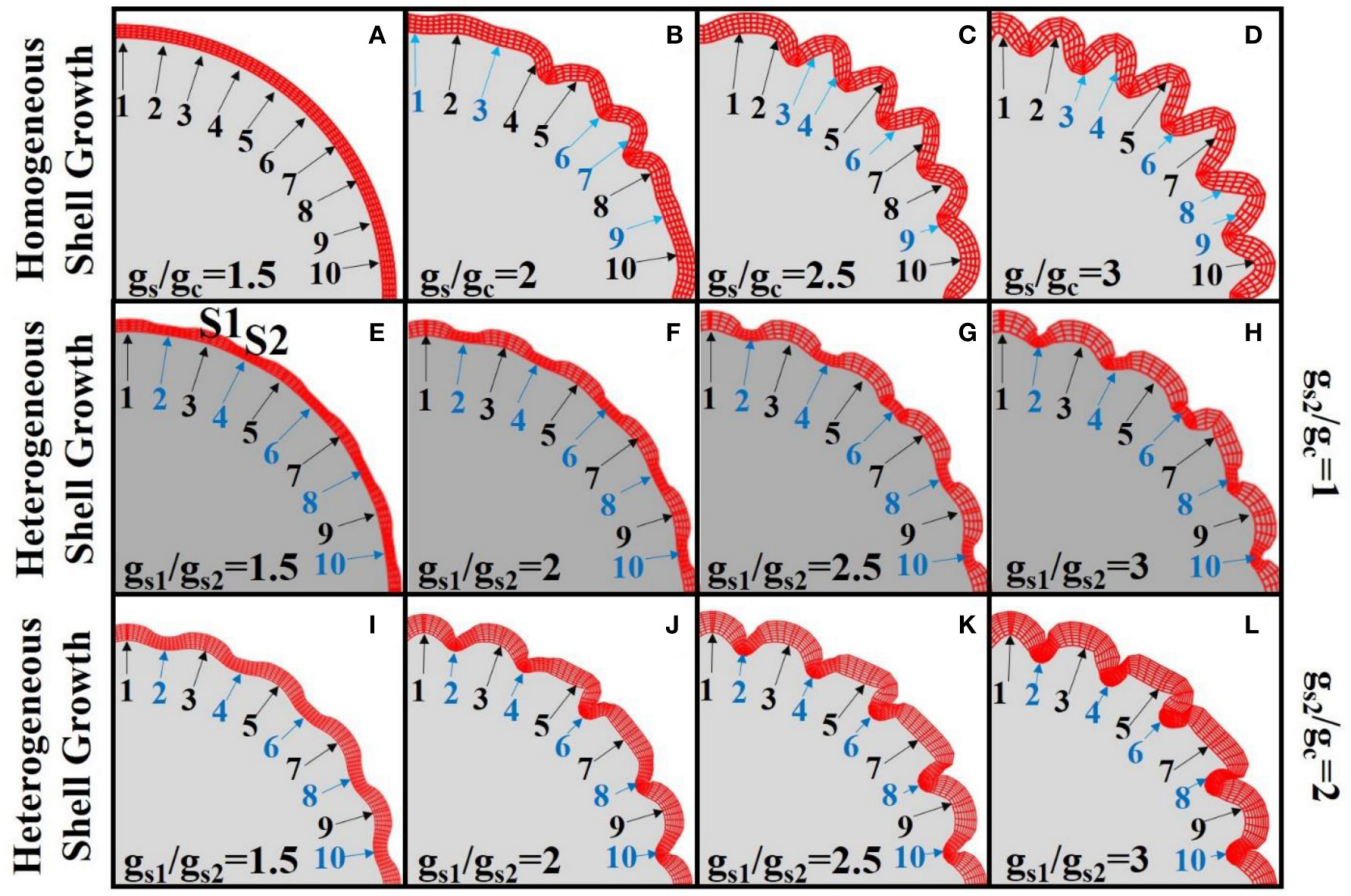
Simulation of cortex growth with different growth speed ratios (g_s_/g_c_) for the 2D core-shell model. Red color mesh depicts the shell of the model while the solid gray part is the core. Numbered arrows indicate corresponding locations on the shell for each image. In the first row **(A–D)**, the growth speeds in shell regions are the same. Different growth speed ratios between shell and core are used in each sub-figure. In the second row **(E–H)**, the shell regions highlighted by the odd numbered arrows grow faster than those highlighted by the even numbered arrows (g_s1_ > g_s2_ = g_c_). In the third row **(I–L)**, the shell regions highlighted by the odd numbered arrows grow faster than those highlighted by the even numbered arrows. The latter ones also have faster growth speed than the core (g_s1_ > g_s2_ = 2g_c_). We use blue arrows and numbers to indicate sulci and black arrows and numbers to indicate gyri. The simulation results shown in each sub-figure are snapshots of the model after growing for the same amount of time.

However, the effect of heterogeneous growth speeds between laminae does not regulate the patterns of radial cortical folding by itself. The upper row of Figure 6 shows the results with a variety of growth ratios (g_s_/g_c_) between the shell and core of the model after the same simulation time. It is observed that, while convolution becomes more elaborate with increasing growth ratios (g_s_/g_c_), the laminar growth speed difference does not regulate the convolution patterns. To better clarify this point, we highlight 10 corresponding locations using numbered arrows on the shells in sub-figures of the first row. The positions on the shell are of blue color if they are located near sulcal fundi and of black color otherwise. It can be observed that consistent radial folding patterns are not always reproduced at the same location when different growth ratios are employed. For example, no sulcus is identified between arrows #1 and #2 in Figure 6C, but a sulcus can be found between them in Figure 6D. Another example is the location of arrow #8, which appeared on a gyrus in Figure 6B, on the gyral wall in Figure 6C, and on the lower part of the gyral wall in Figure 6D.

In contrast to the model with homogeneous growth speed within the shell, we introduce a model with regional growth speed differences in the shell region (the second row and third row of Figure 6). Under the assumption that more neurons migrate to and accumulate in the regions with more RGCs, we initialize the shell by assigning higher growth speeds to certain periodic shell regions (s_1_, highlighted by black arrows) than the others (s_2_, blue arrows highlighted), i.e., g_s1_ > g_s2_. In order to simplify the analysis, for Figures 6E–H) we set the growth speed of s_2_ region as the same as core, i.e., g_s1_ > g_s2_ = g_c_. It is interesting to see that the convex and concave patterns are consistently formed within the s_1_ and s_2_ regions, respectively. Moreover, we explore the relationship between convolution patterns and the shell thickness (Supplemental Figure 4d) on the model in Figure 6H and find that shell thickness decreases with the increase of concavity and the decrease of convexity. This result is consistent with the one based on imaging data shown in Figure 4f and is also in agreement with the results in Figure 5 that convex cortical regions have more neurons, which may elicit a thicker cortex (Hilgetag and Barbas, 2005). Finally, in order to test the robustness of the results, we use another set of configurations, i.e., g_s1_ > g_s2_ = 2g_c_, so that all of the shell regions grow faster than the core (Figures 6I–L). Again, convex and concave patterns are consistently produced in s_1_ regions and s_2_ regions, respectively, demonstrating the robustness of these results.

In summary, consistent and reproducible radially convex and concave convolution patterns of the cerebral cortex are regulated by regional growth heterogeneity.

### Axons serve as regulators of convex radial convolution

In this section, we report the interaction between axonogenesis and gyrogenesis. Two mechanical factors of axons, their mechanical forces and their density, are separately studied and compared with computational models.

#### Imaging data evidence

**Figure 7:**
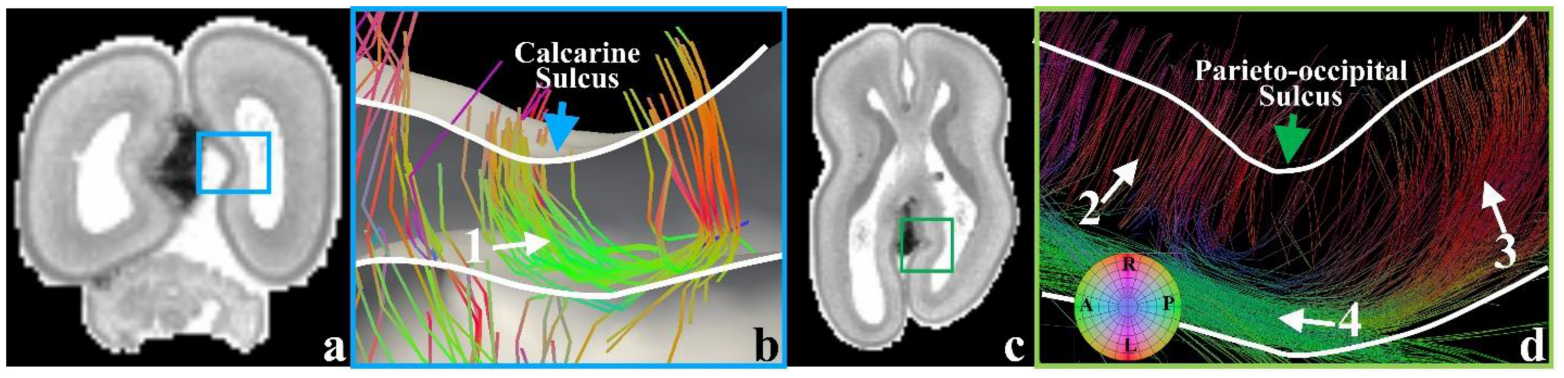
**(a)** A coronal T1-weighted MRI section of the 21 pcw fetal brain. The blue frame highlights the location of the calcarine sulcus; **(b)** Tractography fibers extracted from the calcarine sulcus. Arrow #1 indicates radial structure composed of RGCs and neuronal axons, coursing along the sulcal regions; **(c)** A transverse T1-weighted MRI section of the 21 pcw fetal brain. The green frame highlights the location of the parieto-occipital sulcus; **(d)** Tractography fibers extracted in the parieto-occipital sulcal regions. The green arrow highlights the sulcal fundus location on the cortical surface. Arrow #2 highlights RGCs. Arrow #3 indicates the radial structure, a mixture of RGC and neuronal axon. Arrow #4 highlights the parallel fibers beneath the sulcal fundus. White curves indicate the cortical plate boundaries.

In the later phase of the first stage of mammalian brain development, maturing neuronal axons might be another factor in the generation of radial convolution (Takahashi et al., 2012). During this period, the RGCs begin to diminish in number within the deep sulcal fundi. However, they still persist in gyral regions with the emergence of abundant projection fibers such as neurons in the primary sensory cortical areas projecting back toward the primary sensory thalamic nuclei and long range association fibers, which radiate from one cortical region to another (Takahashi et al., 2012). It has been suggested by a growing number of reports that in either the developing brain (Takahashi et al., 2012) or the adult brain (Budde and Annese, 2013), the terminal ends of those structures are radially concentrated on gyral crests and walls more than sulcal fundi. These observations are reproduced in the 21pcw fetal DTI data (Figure 7). To explore the developing brain, tractography fibers are extracted from the calcarine sulcus and parieto-occipital sulcus of 21 pcw fetal brain DTI data (http://www.brainspan.org). Abundant structures are radially oriented in their neighboring gyral walls (white arrows #1, #2, and #3) and contact with the cortical plate. But they are tangentially oriented in the nadir of the sulcus (white arrow #4), suggesting that very few structures emanate from such cortical plate regions. These radially oriented structures are suggested to be a mixture of both diminishing RGCs and maturing long-range projection neuronal axons (we will use the term “axons” to represent them.) in Takahashi et al. (2012). Similar observation can also be found in a matured canine brain (Supplemental Figure 8). Therefore, in the radial direction, we assume that convex regions could have a positive relationship with density of axonal termini.

#### Computational analyses of axon's role in convex radial convolution

It is still controversial in the literature whether the grown axons are in tension or compression (Van Essen, 1997; Xu et al., 2010; Nie et al., 2012; Wedeen et al., 2012; Budde and Annese, 2013), that is whether gyri are formed by either a “pulling force” or “pushing force” on the axons. Therefore, we set shell, core and fibers in the computational model with different growth speeds so as to mimic axons with different mechanical properties. When fibers grow faster than the shell, they are in compression and provide a “pushing force.” Otherwise, they are in tension and provide a “pulling force” to the location where they connect the shell.

Disregarding all other factors such as the growth speed difference between the shell and core, it is straightforward that when axon is in compression it “pushes” the cortical plate outward to form a “gyrus” and it “pulls” the cortical plate inward to form a “sulcus” when it is in tension (see Supplemental Figure 6 for more details).

**Figure 8:**
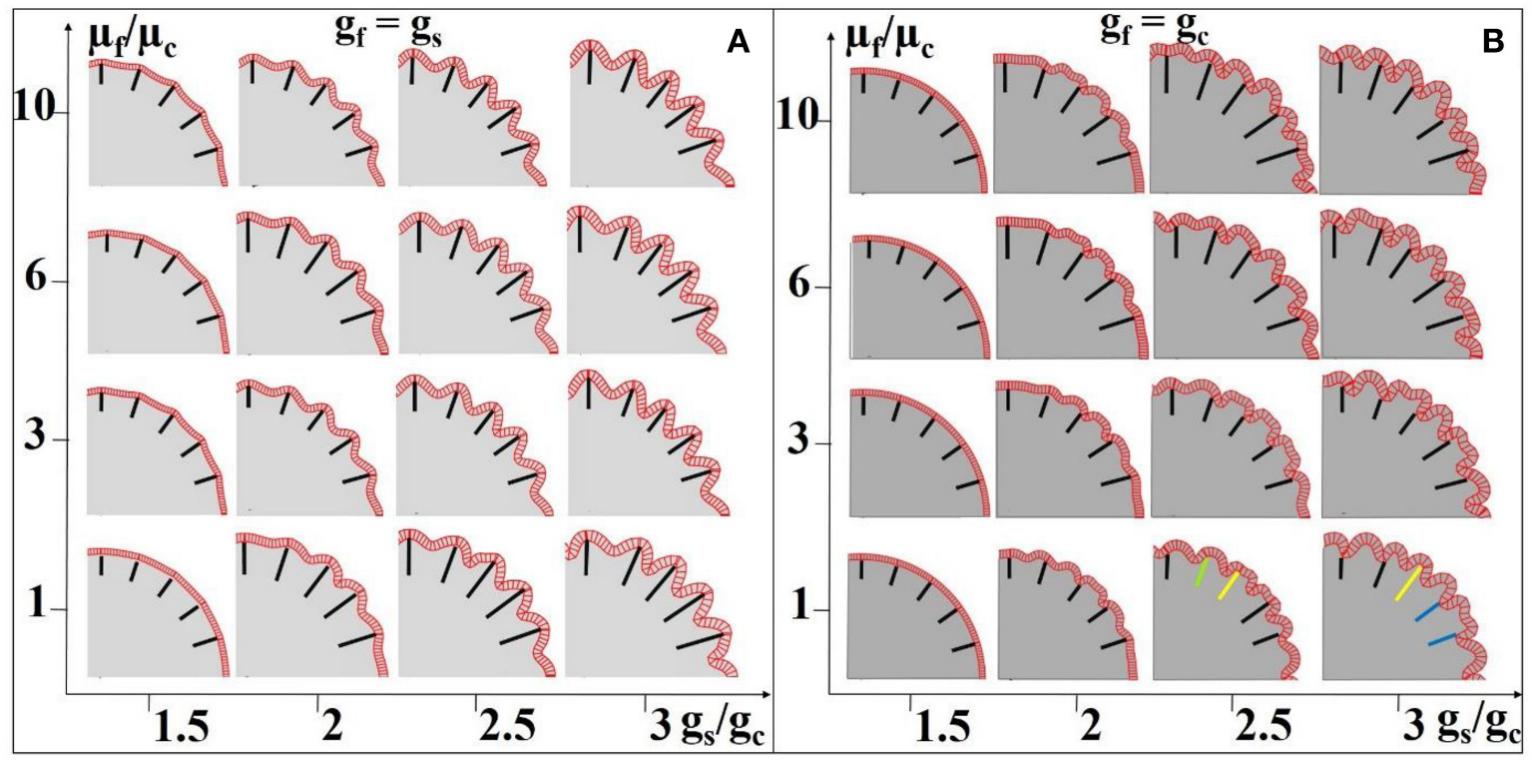
Effect of axon density on the convolution patterns. Black and colored bars indicate regions where axons are denser than elsewhere in the core. The axon density is modeled by fiber stiffness in the computation model. The fibers growth speed is the same as the shell (g_s_) in **(A)** and the fiber growth speed is the same as the core (g_c_) in **(B)**. A variety of growth speed ratios g_s_/g_c_ (x-axis) and stiffness ratios between axons and the core μ_f_/μ_c_ (y-axis) are considered. The stiffness values of the shell and core are the same for all cases. Yellow bars highlight convex convolution patterns, blue bars highlight concave convolution patterns and green bars highlight intermediate patterns.

In addition, we choose different stiffness values for the core and fibers so as to mimic axon bundles with different density values. The results are summarized in Figures 8A,B. The stiffness of the axons (highlighted by black line segments) μ_f_ is equal to or greater than that of the core regions (the stiffness of the shell is set to be the same as that of the core, Xu et al., 2010) μ_c_, suggesting that denser axons emanate from the shell regions they attach to. We set a homogeneous growth speed within the shell in order to eliminate regional differences, but set the growth speed of the shell (g_s_) faster than that of the core (g_c_). As axons emanate from the cortical plate, the growth speed of axons in Figure 8A is the same as that in the shell. It can be observed that convex patterns are consistently generated in the locations where denser axons emanate. For comparison, the growth speed of axons is set to be the same as that of the core and the results are shown in Figure 8B. No consistent relationship between radial convolution patterns and axons' stiffness are found, especially when growth speed ratio g_s_/g_c_ is greater than 2. The radial convolution patterns in the locations where the axons attach to can be either convex/gyral regions (highlighted by yellow bars) or concave/sulcal regions (highlighted by blue bars) or even the intermediate regions/gyral walls (highlighted by green bars). This observation suggests that the radial convolution in Figure 8B is controlled by the growth speed difference between the core and the shell but not axons because their growth speed is the same as the core. In contrast, the stiffness of the axons shows its effect in Figure 8A, suggesting that it plays an active role in the production of gyral patterns, although these axons grow as fast as the shell and provide no active mechanical force. Finally, we measure the shell thickness and curvature on the model in the right-bottom corner of Figure 8A. The concave regions are thinner than the convex regions (Supplemental Figure 4e), which is consistent with the relationship from our imaging data shown in Figure 4f.

In summary, based on imaging data observation, we find that axons have a close relationship with the formation of convex radial convolution. By using computational models, we separately study the effects of mechanical forces due to the growth speed difference and due to axonal density on the convolution patterns, as well as investigate their relationship with convex radial convolution. Compression in axons and large axonal density may each play a positive role in producing convex/gyral convolution patterns.

### Interplay of neurogenesis and axonogenesis acts as a convex (gyral) convolution pattern regulator

In the previous two sections, we separately study the possible roles of neurogenesis and axonogenesis in regulating radial cortical convolution. However, a few problems still remain unclear when only one factor is considered. For example, it is still contentious in the literature whether the axons are in tension or compression, and it is widely reported that denser axons emanate from more convex regions (Nie et al., 2012; Wedeen et al., 2012; Budde and Annese, 2013). If we consider the mechanical force in axons as the single factor, it is difficult to generate convex cortical regions with the axons being under tension (“pulling force”). Therefore, in this section, we extend our study to investigate the joint effect of these two factors.

**Figure 9:**
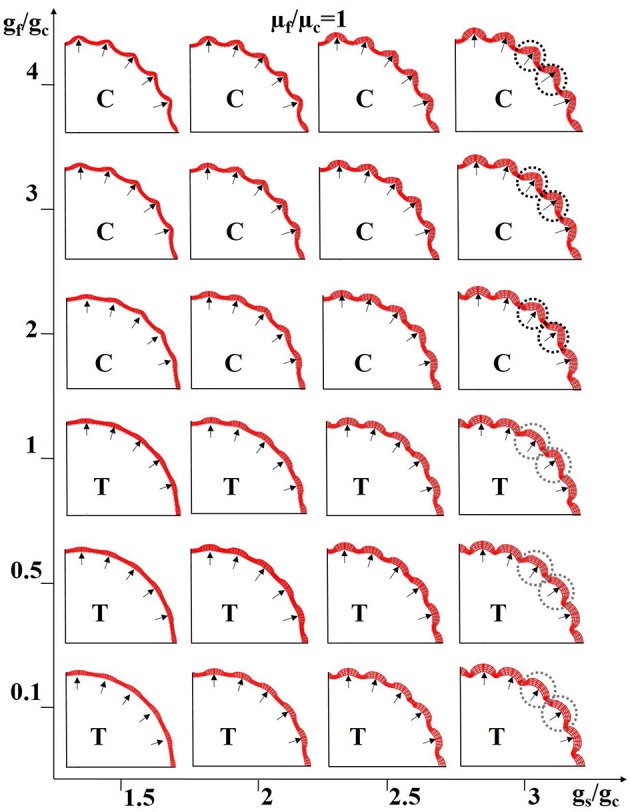
Combined effects of neurogenesis and axonogenesis on the convex convolution patterns of cerebral cortex. Black arrows highlight regions where denser axons are connected; these regions are thus endowed with higher stiffness. The shell growth speed in intermittent sections (near the black arrows) is faster than the other shell regions and core. The growth speed ratio g_f_/g_c_ between axons and the core generally controls the scenarios under which the axons are in tension (T) or compression (C). Black dashed circles and gray ones highlight the typical convex convolution patterns when axons are in compression and tension, respectively. For these experiments the stiffness of the axons is considered to be the same as that of the core.

From Figure 6, it can be seen that a faster growth speed of the cortical plate due to more migrating neurons can lead to the formation of a convex pattern. We assume that more axons will also radially emanate from those convex regions because more neurons will create more axons, and they will have higher possibility to connect to other convex regions that have more neurons than to the relatively neuron-poor concave regions. Based on this assumption, we design the computational models shown in Figure 9 to study the joint effects of these two factors (neurogenesis and axonogenesis) which are controlled by parameters such as the growth speed ratio between the axon and the core and the growth speed ratio of the shell to the core. The black arrows highlight shell regions which have a higher growth speed and thus where denser axons are attached.

To simplify the model configuration, we set the growth speed in the slow-growing shell regions to be the same as that of the core. In this way, the growth ratio g_s_/g_c_ represents both the regional growth speed difference across the shell sections and the radial difference between the fast-growing shell and the core. Also, we utilize the growth ratio between the fiber and the core g_f_/g_c_ to control whether axon is in tension (g_f_/g_c_ <= 1, axon is in tension when g_f_/g_c_ = 1 because g_s_/g_c_ > 1) or compression (g_f_/g_c_ > 1). The black arrows in Figure 9 represent denser axons, the stiffness of which is here the same as that of the core (μ_f_/μ_c_ = 1) in Figure 9. Other results based on stiffness ratios like μ_f_/μ_c_ = 10 are shown in Supplemental Figure 7.

It is not surprising that gyri are formed at locations highlighted by black arrows when axons are in compression (sub-figures marked with “C”) because these shell regions grow faster and the axons also grow faster so as to “push” the shell outwards and both factors play a positive role in the formation of gyri. It is intriguing to note that gyri are also generated at the location of denser axons even when the axons are in tension (sub-figures marked with “T”), if the growth speed in arrow-highlighted shell regions is fast enough to compensate the displacement where the axons have “pulled” the shell inward. Although, convex region can be produced when axons are in either tension or compression, different patterns can still be observed. For example, the gyral crest is relatively sharp if the axon is in compression (black dashed circles) and it is flat when the axon is in tension (gray dashed circles). The shell thickness and curvature are measured on the models in the right-top corner and right-bottom corner of Figure 9. No matter which gyral pattern the model produces, sharp top (Supplemental Figure 4f) or flat top (Supplemental Figure 4g), thicker shells can always be found in gyral regions while thinner ones are seen in sulcal regions, similar to the relationship gathered from the imaging data shown in Figures 4g,h. In summary, the joint effects of neurogenesis and axonogenesis regulate the folding patterns of convex regions. Different convex region patterns might be created by different combinations of effects.

## Conclusion

This paper suggests that the commonly preserved radial convolution seen in the mammalian cortex is regulated by radial structures during the early development stage of the brain. The regionally heterogeneous distribution of RGCs may regulate the locations of gyri and sulci patterns, where the interplay of axonogenesis and neurogenesis may regulate the folding patterns of gyri. These suggestions are supported by a variety of evidence from multiple disciplines, e.g., genetic studies (Stahl et al., 2013; Borrell and Götz, 2014), imaging data analyses in literature (Wedeen et al., 2012; Budde and Annese, 2013) as well as the computational simulations conducted in this work. A joint study of data analysis and computational modeling offers valuable insights to evaluate specific hypotheses of cortical morphogenesis and is helpful for exploring the possible mechanisms of cortical folding.

Undoubtedly, brain development is a series of complicated processes. We only discussed the formation of convolution in the early stage (Figure 1D) of the brain development, during which the relatively less variant primary convolutions are formed in the radial direction. Two processes, i.e., neurogenesis regulated by RGCs and axonogenesis of the long-range projection axons which occur almost concurrently, are assumed to be the critical causes of gyrogenesis. Many other processes like the maturation of short range axons, axon pruning, and axon myelination may also play a critical part in gyrogenesis in the secondary and tertiary stages. For example, short-range axons (U shape axons) were reported (Zhang et al., 2014) to have a close relationship with convolution variations in the tangential direction referring to the circumferential cortical landscape based on a comparison study among mammalian species. However, how and when these processes interactively and dynamically contribute to cortical convolution still remains largely unknown (Van Essen, 1997; Monuki and Walsh, 2001; Grove and Fukuchi-Shimogori, 2003; Sur and Rubenstein, 2005; Rakic, 2006).

Fundamentally, the formation of cortical convolution is regulated by genetic factors. For example, cortical convolution patterns, though varying across subjects, show great correlation between twins (Biondi et al., 1998; White et al., 2002) and even close relatives (Baare et al., 2001; Thompson et al., 2001). However, as a physical identity, the brain is also assumed to develop under the constraints of a series of mechanical forces and physical properties. The genetic factors can only take effect by regulating those processes at the base level. Thus, interpretation of developmental mechanics provides an opportunity for us to bridge the gap between genetic influence and mechanical forces induced phenotypic characterization, and eventually offers insight into both developmental mechanisms as well as possible brain malformation triggers (Dobyns et al., 1993; Raybaud and Di Rocco, 2007).

It is worthwhile to mention that in the computational models proposed in this paper, there are some simplifications and assumptions which impose limitations to the results. First, the gyrification index in the 2D models is smaller than that of a real brain, where it can be up to 3 dimensions (Geng et al., 2009; Tallinen et al., 2014). Second, in our model, the shell and core are assumed to exhibit isotropic behavior whereas in the real brain both gray and white matter show anisotropic behavior (Arbogast and Margulies, 1998). In some studies the anisotropy of the core (white matter) has been modeled by a stretch driven property to mimic the axons' contribution to the deformation of the developing brain (Bayly et al., 2013; Budday et al., 2014), although the assumptions cannot exactly cover the roles of axons and glial cells on the regulation of convolution patterns in the developing cerebral cortex (Takahashi et al., 2012; Borrell and Götz, 2014). It entails a significant amount of research in order to bring proper glial and axonal contribution to mechanical models. Third, in this paper, smooth circular or elliptical initial shapes have been considered as the initial geometry of the developing brain (Bayly et al., 2013; Budday et al., 2014), while the developing brain at early stage is not such a regular shape (Prayer et al., 2006). Therefore, presenting the proper initial geometry may lead to the better depiction of convolution patterns.

## Ethics statement

Fetal brain data from http://www.brainspan.org was used, in which all work was performed according to guidelines for the research use of human brain tissue and with approval by the Human Investigation Committees and Institutional Ethics Committees of each institute from which samples were obtained. Appropriate written informed consent was obtained and all available non-identifying information was recorded for each specimen.

## Author contributions

MR: conducted computational modeling experiments and manuscript drafting; TZ: conducted imaging data analysis and manuscript drafting; HC and YL helped with imaging data analysis and manuscript editing; SP: helped with animal histology data acquisition and analysis; YZ: conducted data processing; Theory and methods were originally proposed by LG, XH, XW, and TL. They also helped with manuscript editing.

### Conflict of interest statement

The authors declare that the research was conducted in the absence of any commercial or financial relationships that could be construed as a potential conflict of interest.

## Footnotes

^1^*See “Developing Human Brain Imaging” document in* *http://help.brain-map.org//display/devhumanbrain/Documentation*

^2^*See “Reference Atlases” document in* *http://help.brain-map.org//display/devhumanbrain/Documentation*.
