# Supplementary material for: Radial Structure Scaffolds Convolution Patterns of Developing Cerebral Cortex
Source: Front Comput Neurosci. 2017 Aug 15;11:76. doi: 10.3389/fncom.2017.00076 (PMC5559440; doi:10.3389/fncom.2017.00076)
Supplement: Supplementary file 1 [file Presentation1.PDF]

Supplemental Materials

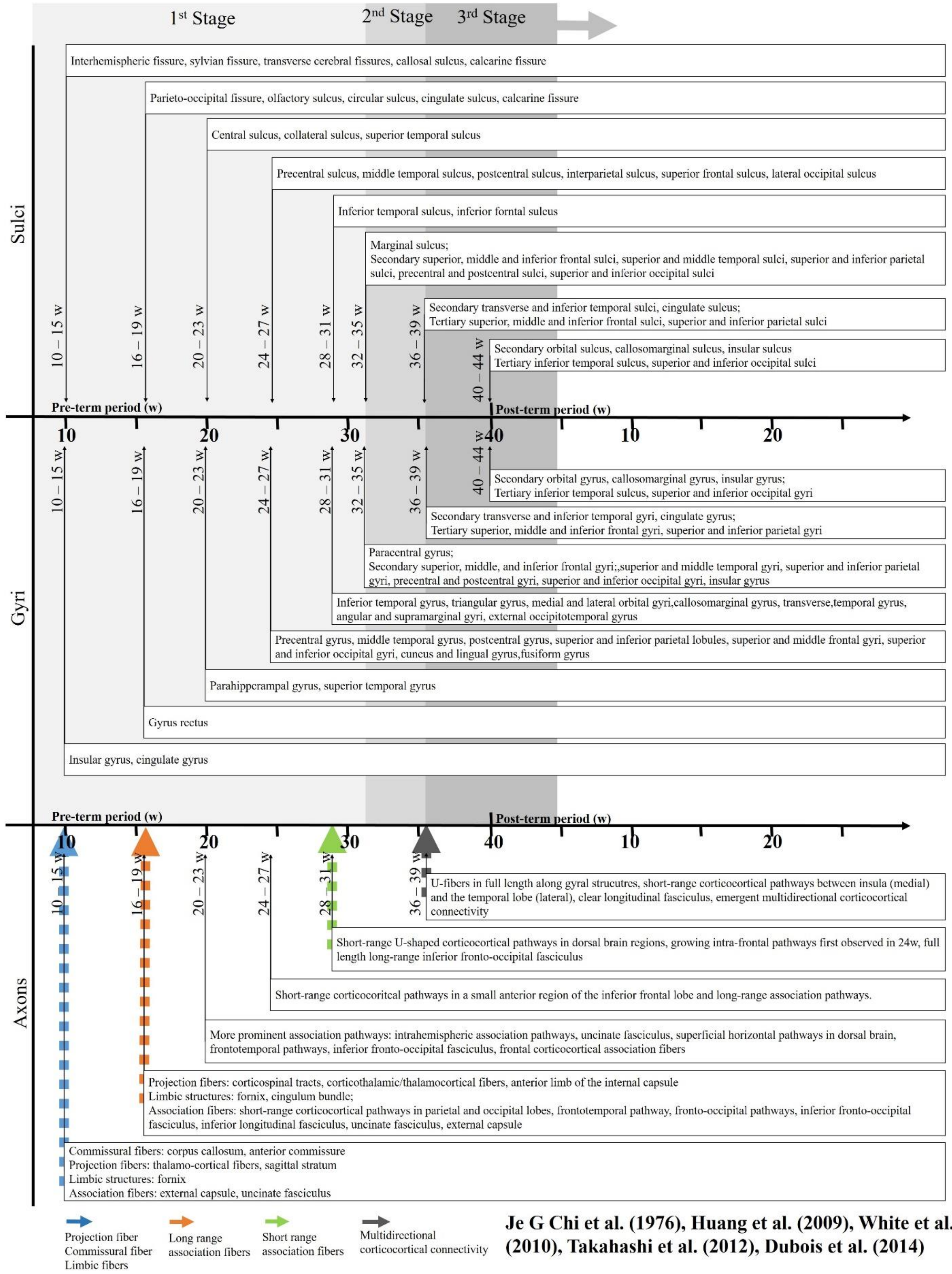

Je G Chi et al. (1976), Huang et al. (2009), White et al. (2010), Takahashi et al. (2012), Dubois et al. (2014)

Figure 1. Time evolution of gyrogenesis and axonogenesis of the human brain. The time segments refer to the work by White et al., 2010. The dashed arrows in the fiber panel indicate the boosting time point of the special type of axons. Key references are listed in the right bottom of the figure.

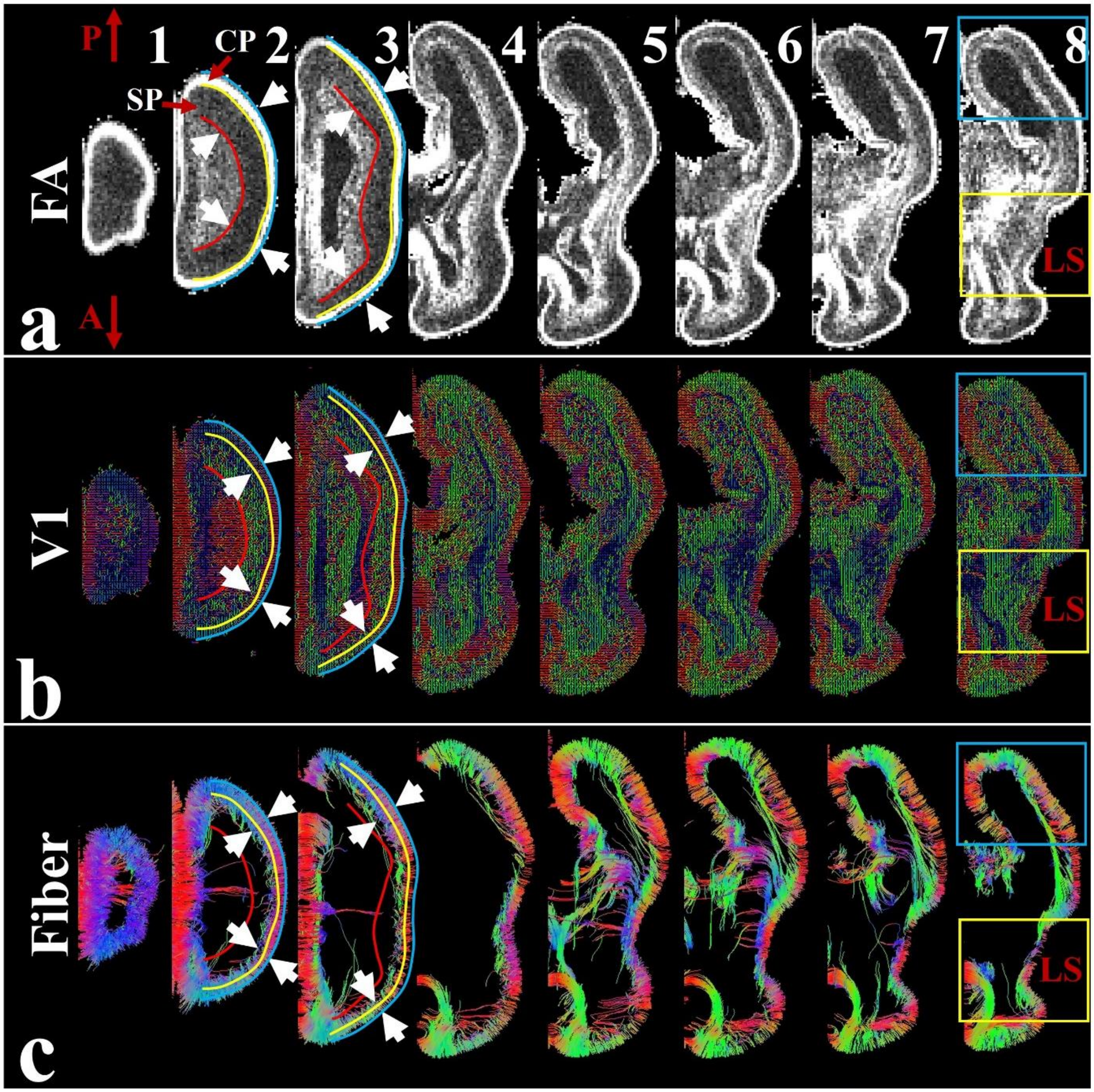

Figure 2. (a) Eight transverse sections extracted from the FA map; (b) eight transverse sections extracted from the V1 map; (c) tractography fibers passing through the eight sections; white arrows in (a) indicate the thickness of the SP+CP; arrows in (b) and (c) indicate the length of radial structures. Regions

highlighted with blue/yellow frames are detailed in Figure 4(a)/(b) in the main text. White paired arrows indicate the thickness of SP+CP in the FA map and V1 map and the length of reconstructed radial structures.

Computational model

Computational modeling has been emerging as a powerful technique to validate or verify the results from experiments, in which finite element analysis has offered valuable insights into the growth, morphology and function of the brain. For example, results showed that tangential cortical expansion itself might be heterogeneous in both time and space (Xu, et al., 2010); faster cortical expansion leaded to shorter gyral wavelength; and no inner or outer (skull) constraint was needed to produce folding (Bayly, et al., 2013). Soft material without a hard skin by compression beyond the critical value leads to the formation of creases with sharp edges. One of the main characteristics of creases is the development of self-contact phenomenon after instability (Jin, et al., 2011; Cao, et al., 2012; Razavi, et al., 2015). This phenomenon can be expected in growing cortex as compressive stress in outer layer of cortex increases due to growth, triggers instability, and form creasing. Figure 3 shows the morphological evolution of the growing core-shell tissue with the von Mises stress distribution at different times. It is inferred from the Figure3(a) in this supplemental material that stress distributions are uniform for both the core and shell of the model and the compressive stress in outer surface of the shell is not large enough to trigger the instability. With the continuation of growth, the compressive stress in the shell loses its uniformity in Figure 3(b). Beyond the critical point of the compress stress in the shell the model becomes instable and reaches to a new stable configuration by formation of creases as depicted Figure 3(c).

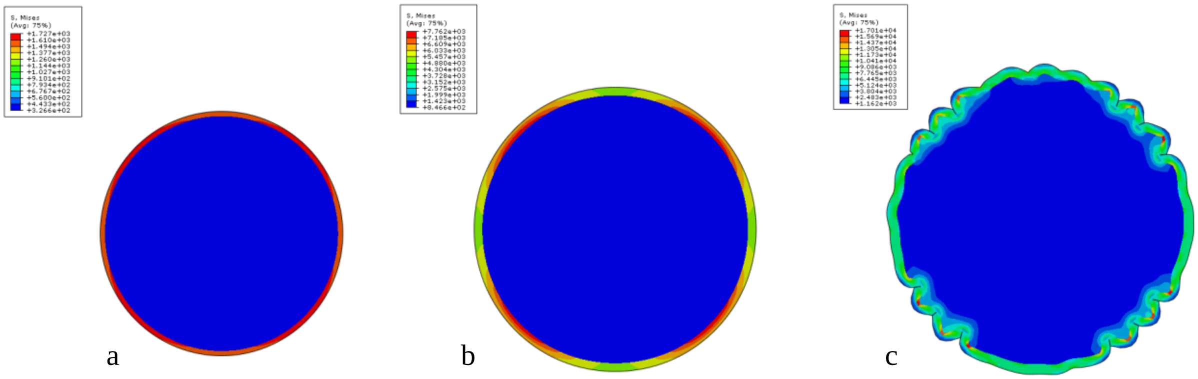

Figure 3. Morphological evolution of the growing brain with von Mises stress distribution at different simulation time (a) time = 0.4, (b) time = 0.6, and (c) time = 0.7 for $g_s=3g_c$. Shear modulus for both core and shell sections is[9] $\mu = 2$kPa and stress counter unit is Pa.

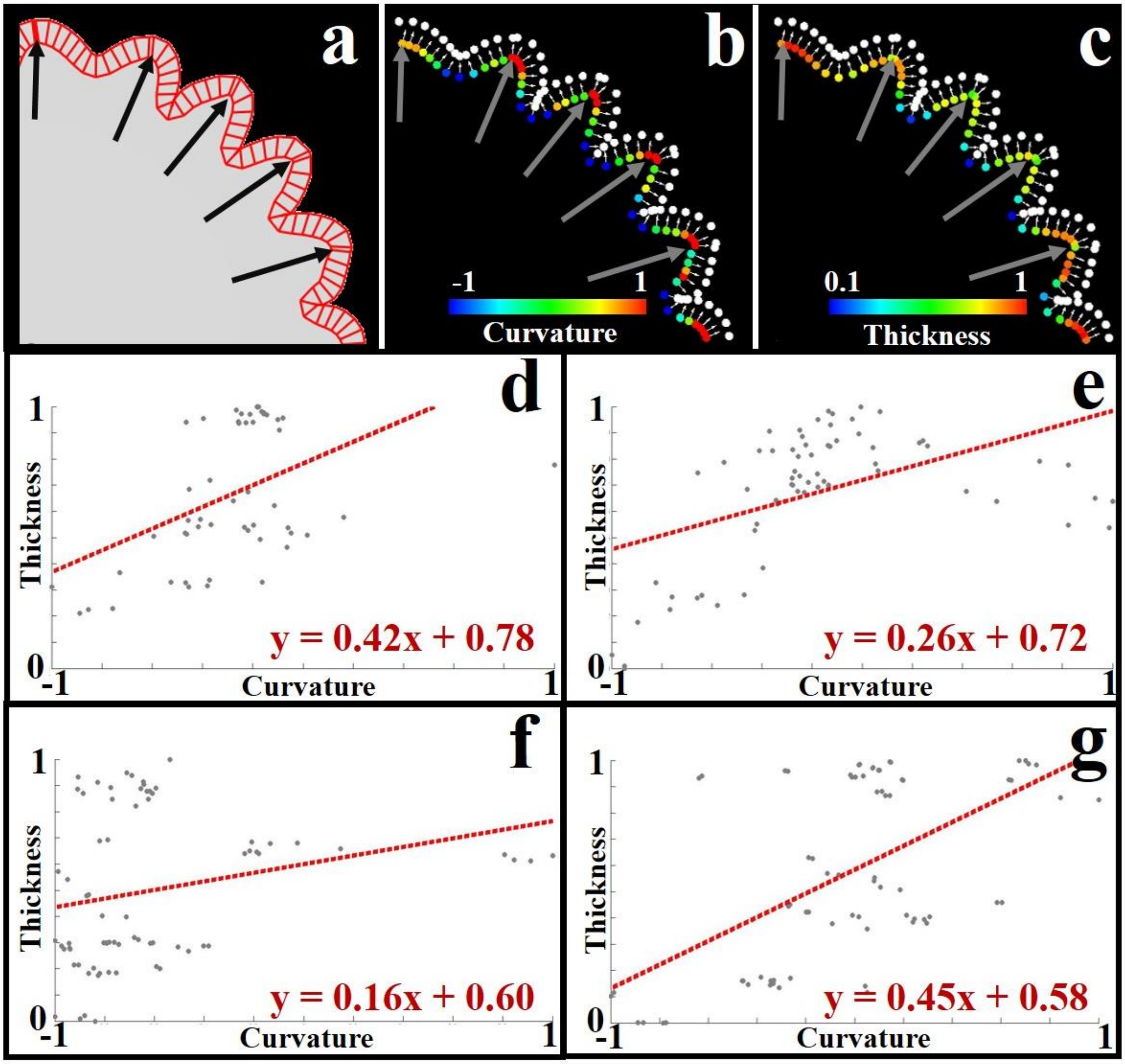

Figure 4. The relationship between shell thickness and folding pattern (curvatures) on the computational models. (a)-(c) illustrate the method used to obtain curvature and thickness for the vertices on shell. Firstly, vertices on the inner layer (color bubbles) and the outer layer (white bubbles) of the shell are respectively extracted. Secondly, 2D linear curvature is computed for each vertex on the inner layer shown in (b). Thirdly, normals, white arrows between two layers, are computed for all vertices on the inner layer of the shell. Then, the normals are extended until they reach the out layer and the length of the segments of the normals between two layers is used as thickness shown in (c). The model used in (a)-(c) as illustration is the right-bottom model in Figure 8(a) in the main text. Black/gray arrows indicate where

axons are applied. Thickness of the shell is shown against the curvature in the scatter plots in (d)-(g). The thickness and curvature in (d)-(g) are respectively extracted from Figure 4(h), right-bottom corner of Figure 8(a), right-top corner and right-bottom corner of Figure 9 in the main text. 1st order linear trend is estimated and shown in red dashed line.

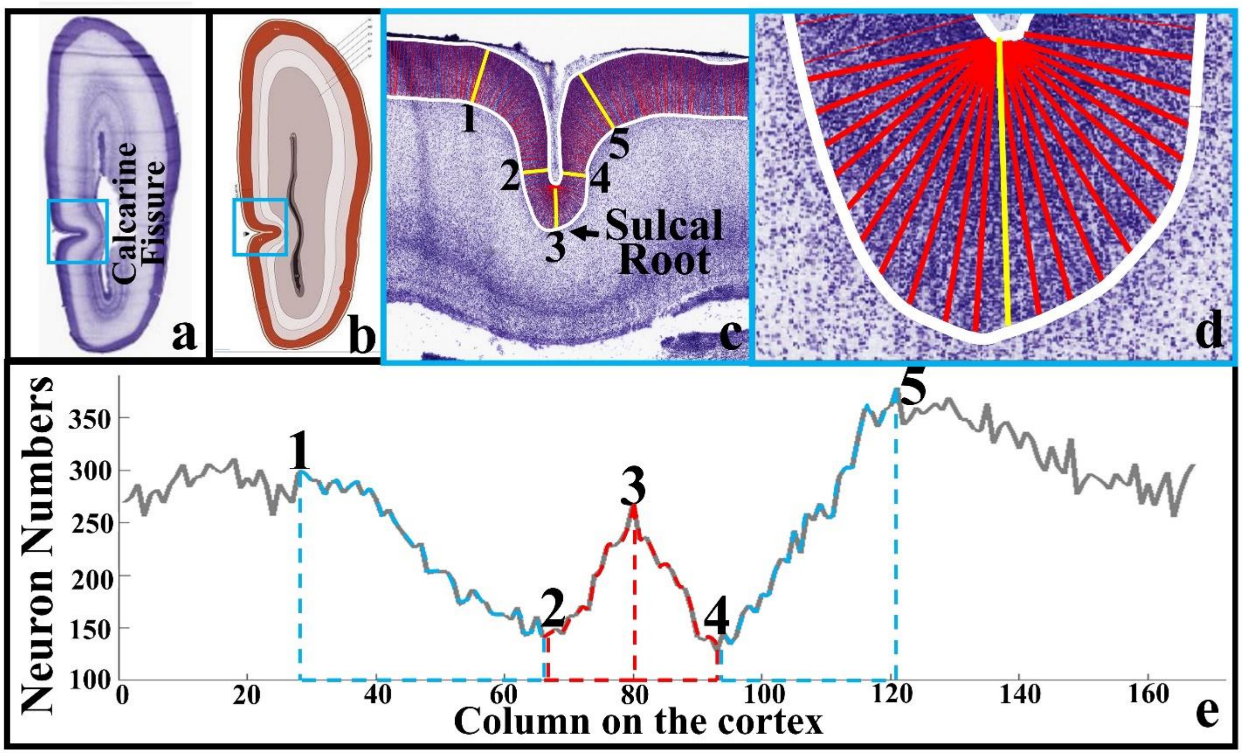

Figure 5. (a) A slice from a 21 pcw fetal brain sectioned in the coronal plane and stained for Nissl; (b)atlas of the slice in (a); (c) The boundary of cortical plane (white curves) were manually depicted. Columns in red and yellow colors were used as neuron number counting boxes. The columns are perpendicular to the upper boundary. The sizes of the counting boxes are 40 μm × height of the column; (d) the settings of columns in (c); (e) Neuron numbers in each column in (c). Numbers highlight some columns with local minimal or maximal neuron numbers. The corresponding locations of those columns can be found in (b). The areas of red and blue dashed frames indicate the rough total neuron numbers in regions on cortical plane covering several columns.

Methods:

In order to better study the relationship between cortical plate thickness and neuron number, we adopt cell identification methods in Arteta, et al., 2012 to count the numbers of neurons in two specific transverse planes of a 21 pcw fetal brain stained for Nissl.

The cell identification methods work within a machine learning scheme. The entire plane was divided into 128×128 blocks. Ten randomly selected blocks were used as training samples, on which we manually identified neurons and labeled the locations of neuron centers. Those training blocks with center location labels were used as inputs to the cell identification methods. A model was learnt from the training blocks and applied to all other ones defined as testing blocks to have the neuron center locations predicted.

Ten testing blocks were randomly selected and have the neurons manually identified. They were used as ‘ground truth’ to evaluate the performance of the cell identification methods. Generally, 88.30% manually labeled neurons were predicted by the methods. Among all the predicted neuron centers, only 9.78% were found located in the non-neuron regions. Those results suggest the methods are effective and have preferably high neuron identification accuracy.

We do the counting as what they did in previous reports that count the number of neurons within a thin column, as highlighted by red color bins. The column goes nearly straight down from upper boundary of cortex to the lower one. The widths of the columns are the same, but the lengths vary and are proportional to the thickness of cortex.

The right side figure shows the neuron numbers from those columns. Some critical ones are highlighted by numbers.

As we can see that #3 column locates in the very bottom of sulcus. Because it has relatively thicker cortex than adjacent sulcal regions (e.g., #2 and #4 columns), it has more neurons. It adjacent sulcal columns have the lowest neuron numbers in this slices. As it is moved to #1 and #5 column, which are located in the 'gyrus' (or at least would like to become a gyrus), the neuron numbers collectively go higher till reach the peak values at #1 and #5.

Although #3 column has relatively more neurons than other sulcal regions, it is still lower than #1 and #5. If we consider the neuron numbers in regions, neuron numbers in the 'sulcal regions' (columns between #2 and #4, the areas of red dashed polygons in the right figure) will be definitely lower than other 'gyrus-like' regions (columns between #1 and #2, and between #4 and 5, the areas of black dashed polygons in the right figure).

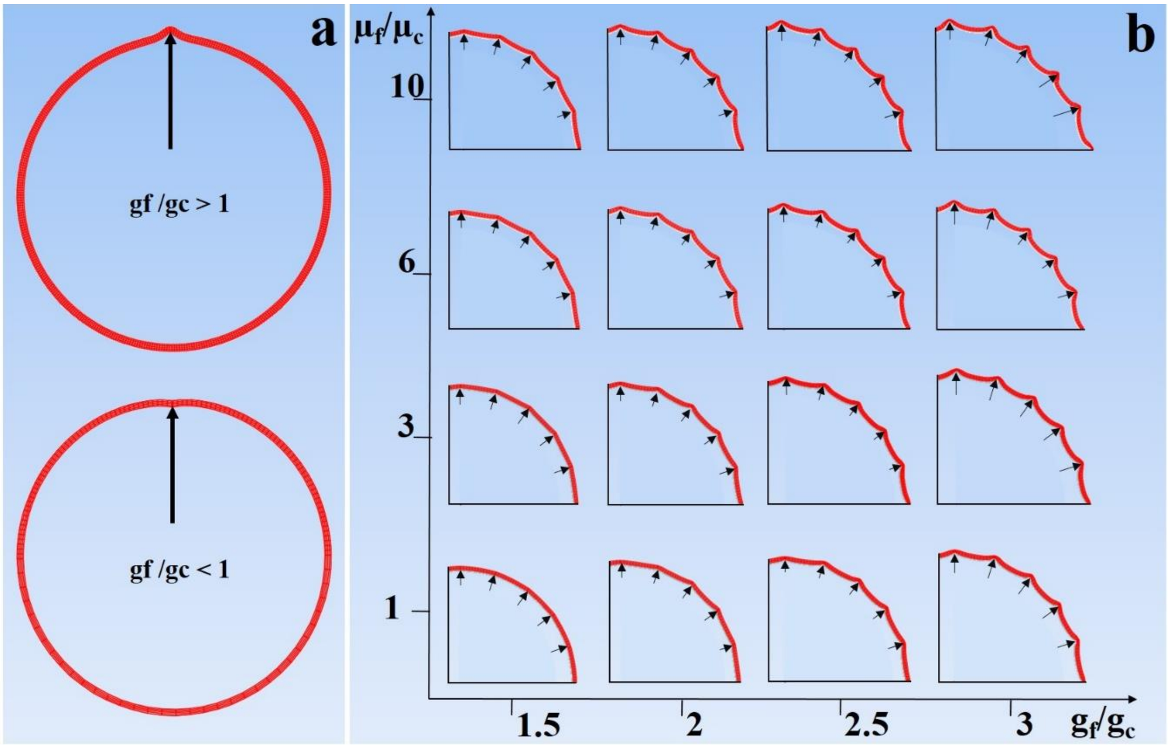

Figure 6. (a) Axons are in compression (top) and in tension (bottom), as is controlled by setting different growth speed ratios between the core (g_c) and axon (g_f). That is $g_f/g_c > 1$ for the top one and $g_f/g_c < 1$ for

the bottom one. The growth speed of core g_c is equal to the shell g_s. The stiffness values of axon, core and shell are the same. Black lines represent axons. (b) Detailed studies on the effect of axons on convolution when they are in compression. X-axis represents the growth speed ratios g_f/g_c. The stiffness values of axons, shell and core are the same. Black arrows indicate where denser axons are applied.

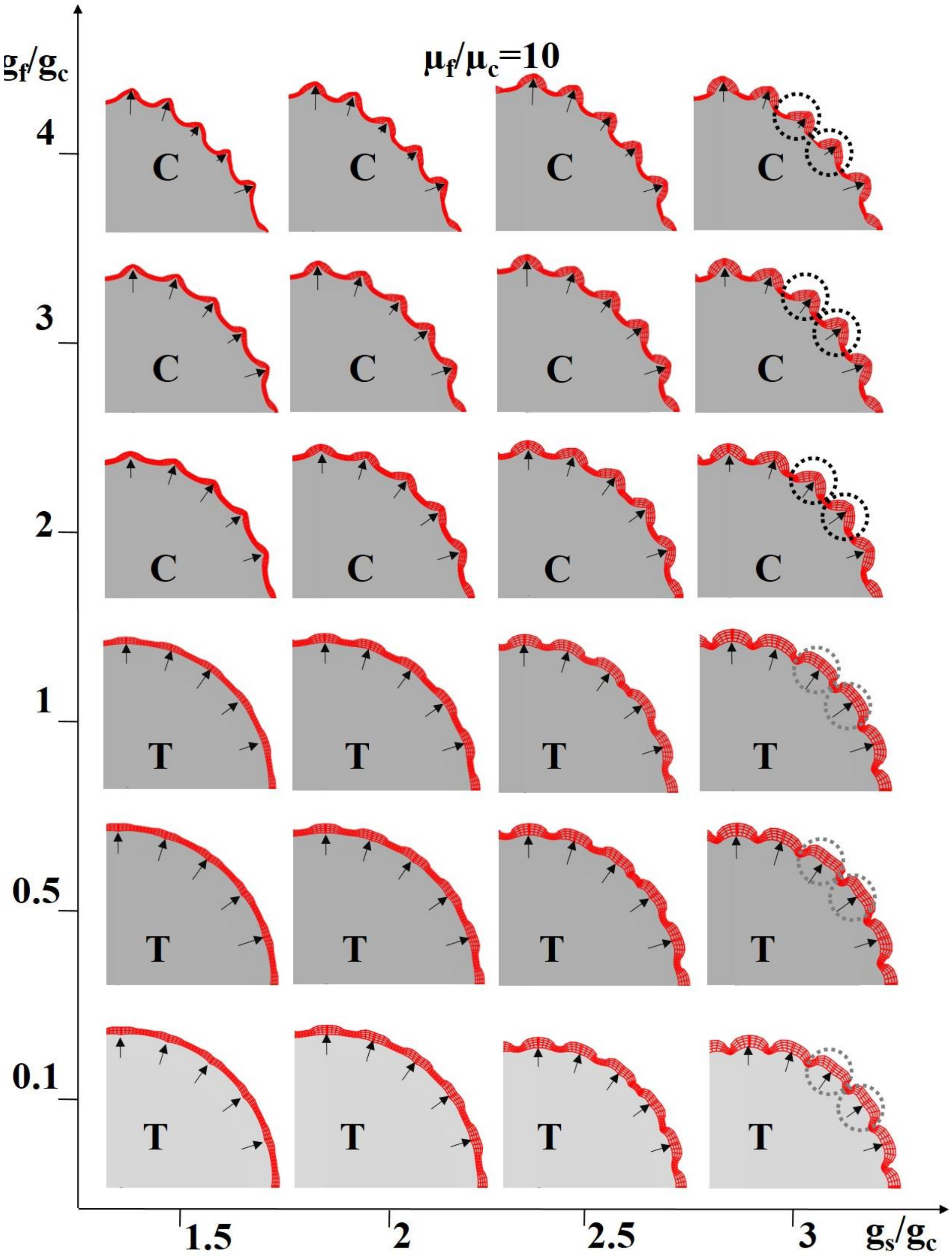

Figure 7. Combined effects of neurogenesis and axonogenesis on the convex convolution patterns of cerebral cortex. Black arrows highlight regions where denser axons are connected. So the regions the black arrows overlapped are endowed with higher stiffness. In these regions, shell growth speed in intermittent sections is faster than the other shell regions and core. Growth speed ratio g_f/g_c between

axons and core generally controls the scenarios under which the axons are in tension (T) or compression (C). Black dashed circles and gray ones respectively highlight the typical convex convolution patterns when axons are in compression and tension. The experiments are conducted when the stiffness of the axons is the same as that of the core.

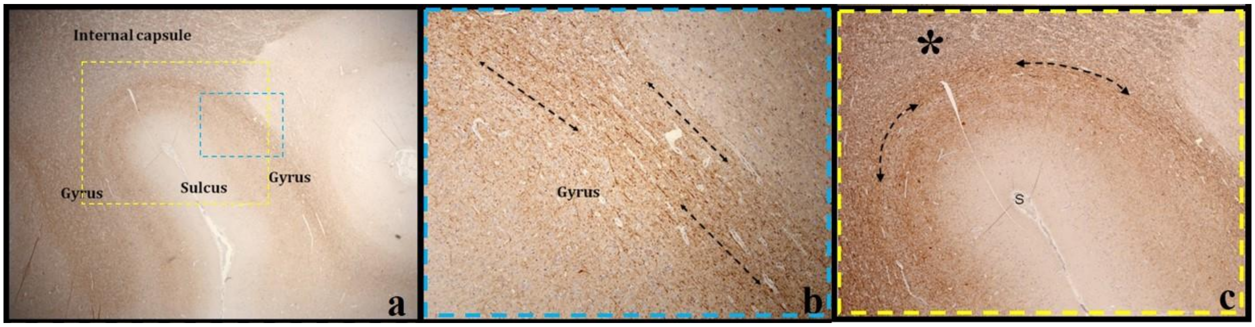

Figure 8. (a) A section of canine brain stained for neuronal axons. The section covers a sulcus in the center and parts of two neighboring gyri; (b) A zoomed in view of the gyral bank region highlighted by the blue frame in (a); (c) A zoomed in view of the sulcal region highlighted by the blue frame in (a); Dashed curves in (b) and (c) illustrate the estimated orientation of stained axons. The asterisks in (c) highlight the region of the nadir of the sulcus. The slice was extracted from parietal lobe of an adult canis lupus familiaris.

References

Xu, G. et al. Axons pull on the brain, but tension does not drive cortical folding. Journal of biomechanical engineering 132, 071013 (2010).

Bayly, P., Okamoto, R., Xu, G., Shi, Y. & Taber, L. A cortical folding model incorporating stress-dependent growth explains gyral wavelengths and stress patterns in the developing brain. Physical biology 10, 016005 (2013).

Rodriguez, E. K., Hoger, A. & McCulloch, A. D. Stress-dependent finite growth in soft elastic tissues. Journal of Biomechanics 27, 455-467, doi:http://dx.doi.org/10.1016/0021-9290(94)90021-3 (1994).

Cao, Y. & Hutchinson, J. W. From wrinkles to creases in elastomers: the instability and imperfection-sensitivity of wrinkling. Proceedings of the Royal Society A: Mathematical, Physical and Engineering Science 468, 94-115 (2012).

Razavi, Mir Jalil, and Xianqiao Wang. "Morphological patterns of a growing biological tube in a confined environment with contacting boundary." RSC Advances 5.10 (2015): 7440-7449.
